# Akt isoform specific effects in ovarian cancer progression

**DOI:** 10.18632/oncotarget.11204

**Published:** 2016-08-11

**Authors:** Nicolle M. Linnerth-Petrik, Lisa A. Santry, Roger Moorehead, Manfred Jücker, Sarah K. Wootton, Jim Petrik

**Affiliations:** ^1^ Department of Pathobiology, Ontario Veterinary College, University of Guelph, Guelph, ON, Canada; ^2^ Department of Biomedical Sciences, Ontario Veterinary College, University of Guelph, Guelph, ON, Canada; ^3^ Center of Experimental Medicine, Institute of Biochemistry and Signal Transduction, University Medical Center Hamburg-Eppendorf, Hamburg, Germany

**Keywords:** ovarian cancer, Akt isoforms, Akt inhibitors, tumor development

## Abstract

Ovarian cancer remains a significant therapeutic problem and novel, effective therapies are needed. Akt is a serine-threonine kinase that is overexpressed in numerous cancers, including ovarian. Mammalian cells express three Akt isoforms which are encoded by distinct genes. Although there are several Akt inhibitors in clinical trials, most indiscriminately target all isoforms. Current *in vitro* data and animal knockout experiments suggest that the Akt isoforms may have divergent roles. In this paper, we determined the isoform-specific functions of Akt in ovarian cancer cell proliferation *in vitro* and in ovarian cancer progression *in vivo.* For *in vitro* experiments, murine and human ovarian cancer cells were treated with Akt inhibitors and cell viability was assessed. We used two different *in vivo* approaches to identify the roles of Akt isoforms in ovarian cancer progression and their influence on the primary tumor and tumor microenvironment. In one experiment, wild-type C57Bl6 mice were orthotopically injected with ID8 cells with stable knockdown of Akt isoforms. In a separate experiment, mice null for Akt 1-3 were orthotopically injected with WT ID8 cells (Figure 1). Our data show that inhibition of Akt1 significantly reduced ovarian cancer cell proliferation and inhibited tumor progression *in vivo*. Conversely, disruption of Akt2 increased tumor growth. Inhibition of Akt3 had an intermediate phenotype, but also increased growth of ovarian cancer cells. These data suggest that there is minimal redundancy between the Akt isoforms in ovarian cancer progression. These findings have important implications in the design of Akt inhibitors for the effective treatment of ovarian cancer.

## INTRODUCTION

Ovarian cancer is the fourth most common cancer in women and is the most lethal gynecologic malignancy [1]. Epithelial ovarian cancer (EOC) accounts for 90% of ovarian cancer cases. The high mortality rate of EOC is due to the lack of early detection and resistance to therapy at advanced stages. Due to the lack of early detection, the 5year survival rate drops from 90% if detected early to 14-20% for late stage detection [2].

At advanced stage EOC, women typically have large primary ovarian tumors, numerous metastatic lesions throughout the pelvis and abdomen, and accumulation of abdominal ascites. Women diagnosed with advanced stage EOC will usually undergo an initial surgical debulking, followed by a chemotherapy regimen consisting of carboplatin and taxol. Unfortunately, many women will develop resistance to this chemotherapy approach and aggressive disease progression will recur [3]. Given these limitations, there has been much interest in “tailored therapies” that target key signaling molecules commonly associated with this disease. Among the growth promoting signaling cascades, the phosphatidylinositol-3-kinase (PI3K)/Akt pathway is of interest [4]. Akt is a pivotal signaling molecule in many mammalian processes including cell growth, proliferation, survival and metabolism and therefore may be an effective target in the treatment of advanced stage EOC.

A previous study using ovarian cancer cell lines and primary ovarian carcinomas showed that Akt was upregulated in 58% of invasive ovarian carcinomas and all ovarian cancer cell lines used in the study had elevated expression of Akt [5]. While total Akt is often measured, humans and rodents express three different Akt isoforms, Akt1 (PKBα), Akt2 (PKBβ) and Akt3 (PKBγ), encoded by three separate genes. The three isoforms share approximately 80% amino acid sequence identity and have been thought to have similar functions in vitro [6]. However, recent studies *in vivo* suggest that the different Akt isoforms may have opposing functions. Knockout mice for specific Akt isoforms display distinct phenotypes, Akt1^−/−^ mice display impaired overall growth [7], Akt2^−/−^ mice display insulin resistance similar to type 2 diabetes [8], while Akt3^−/−^ mice are reported to have a reduced brain size [9, 10]. Double knockout mice have been generated to identify roles of isoform combinations in development and homeostasis. Mice with deletions of Akt1/2 die in the early postnatal period, while Akt 1/3 knockout mice die in utero [11]. Akt2/3 knockout mice are growth impaired, with dysregulated glucose metabolism [12]. Recent in vivo studies, have also reported isoform specific functions in tumor initiation, development and maintenance [13–15]. In mammary tumor mouse models MMTV-neu and MMTV-PyMTV, ablation of Akt1 was shown to delay mammary tumor formation, but had no effect on metastasis [16]. Conversely, Akt2 ablation dramatically accelerated the development of mammary adenocarcinomas in both models. In the mammary tumor mouse model MTB-IGF-IR loss of Akt1 or Akt2 delays mammary tumor onset and suppresses growth [17]. A recent study using a viral oncogene-induced mouse model for lung tumorigenesis demonstrated that Akt1 ablation significantly delays lung tumor initiation, whereas Akt2 deficiency dramatically accelerates tumorigenesis [13]. Akt 3 null mice had a small, but not significant stimulatory effect on tumor growth and progression [13]. TCGA analysis has shown that the Akt pathway is dysregulated in more than 30% of tumors from patients with serous ovarian cancer, and that isoform-specific inhibition of members of the Akt pathway may be a successful therapeutic approach [18]. Given the diverse roles of the Akt isoforms in different types of cancer, we tested the hypothesis that Akt isoform-specific ablation in mouse epithelial ovarian cells (ID8) will have diverse effect on tumor size, survival and metastasis in a wild-type orthotopic syngeneic C57Bl/6 mouse model that replicates high grade serous ovarian carcinoma [19] and that Akt isoforms in the tumor microenvironment contribute differently to tumor progression.

The data from this study have identified Akt isoform-specific effects on ovarian cancer progression. Based on the divergent, isoform-specific effects of Akt signaling in ovarian cancer, the validity of using pan-Akt inhibitors as an anti-cancer strategy is in question.

Our results demonstrated Akt isoform-specific alterations in tumor cells and within the host tumor microenvironment had divergent effects. Within the ID8 tumor cells, knocking down Akt1 resulted in a decrease in tumor size and metastasis 60 days post tumor induction and an increase in survival time. Conversely, tumor cell Akt2 knockdown resulted in significantly increased tumor size, metastasis and decreased survival time. Knocking down the Akt3 isoform moderately increased tumor size, metastasis and survival time compared to ID8 non-target and wild-type tumors. Similar results were seen when the Akt isoforms were altered in the tumor microenvironment. When wild-type ID8 tumor cells were implanted in Akt 2^−/−^ mice, the result was larger tumors and decreased survival time, while ablation of Akt 1 in the tumor microenvironment had an inhibitory effect on tumor size, with no significant change in survival. Thus it appears that isoform-specific Akt signaling regulates tumorigenesis and disease progression with the tumor cells directly, as well as within the tumor microenvironment. An appreciation of the isoform-specific effects of Akt could lead to more effective therapeutic interventions that would target ovarian tumor cells and the ovarian tumor microenvironment.

## RESULTS

### Akt isoform specific knockdown affects tumor growth and metastasis in an orthotopic, syngeneic mouse model of epithelial ovarian cancer

Using lentiviral vectors to deliver shRNAs; Akt 1, Akt 2, and Akt 3 isoforms were successfully knocked down in murine ID8 ovarian cancer cells (Figure 2a and 2b). Knockdown of individual Akt isoforms had differing effects on ID8 cell doubling times. Compared to WT or non-target (NT) controls, knockdown of Akt1 prolonged ID8 cell division, while conversely Akt2 inhibition accelerated ID8 cell doubling rates (Figure 2c). Knockdown of Akt3 had an intermediate effect and resulted in cell doubling similar to controls (Figure 2c). These cells were then injected orthotopically under the ovarian bursa of syngeneic C57BL/6 mice. Tumors were allowed to progress without intervention until 60 days post tumor induction at which time they were sacrificed and tissues collected and analyzed. Injection of Akt1 knockdown (KD) ID8 cells resulted in tumors that were significantly (p<0.01) smaller than any of the other groups (Figure 2d). Conversely, Akt2 KD cells generated the largest (p<0.05) tumors and Akt3 KD cells developed tumors that were larger (p<0.05) than control and Akt1 KD tumors and smaller (p<0.05) than Akt2 KD tumors (Figure 2d). At 60 days post tumor induction, mice were also evaluated for metastatic abdominal spread of disease. Akt1 KD mice had the fewest metastatic abdominal tumors, while both Akt2 KD and Akt3 KD mice had greater metastatic disease than control mice (Figure 2e). Tumors collected at 60d post tumor induction were analyzed for the incidence of cell proliferation, apoptosis, and the extent of tumor vascularization. Tumor sections were immunostained for Ki67 to quantify changes in tumor cell proliferation. Tumors generated with Akt1 KD ID8 cells had significantly (p<0.05) lower tumor cell proliferation compared to control or Akt2 or 3 KD tumors (Figure 3a). Conversely, tumors developed from Akt2 KD ID8 cells had the highest (p<0.05) tumor cell proliferation, while Akt3 KD tumors had intermediate proliferation (Figure 3a). Akt1 KD had the highest (p<0.05), while Akt2 KD had the lowest (p<0.05) incidence of tumor cell apoptosis (Figure 3b), while Akt3 KD tumors had less apoptosis than Akt1 KD tumors, but more than Akt2 KD (Figure 3b). To determine tumor vessel density, the number of CD31-positive blood vessels visible per field of view were calculated. Akt1 KD mice had the lowest (p<0.05) tumor microvessel density, while tumors generated with Akt2 KD cells had the highest density of blood vessels (Figure 3c). Tumors from Akt3 KD ID8 cells again had an intermediate level of vascularization, compared to Akt1 KD and Akt2 KD tumors (Figure 3c). Western blot analysis was performed on lysates from tumors generated from control and Akt1, Akt2, and Akt3 knockdown ID8 cells. Akt1 KD tumors had reduced expression of members of the VEGF family, and higher levels of the pro-apoptotic proto-oncogenes Bad and Bax (Figure 4). Conversely, tumors from Akt2 KD ID8 cells had increased expression of VEGF and its receptor VEGFR2 and reduced expression of pro-apoptotic Bax (Figure 4). While Akt2 and Akt3 KD tumors expressed less Bad than Akt1 KD tumors, they had higher levels of the pro-apoptotic protein compared to controls (Figure 4).

**Figure 1:**
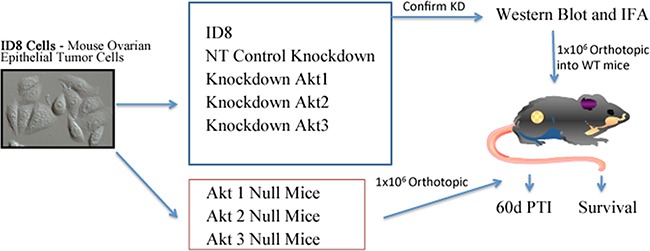
Experimental Design Two *in vivo* models were utilized to determine the Akt isoform specific roles in ovarian tumor progression. In one experiment, mice were subjected to orthotopic injection of 1 × 10^6^ ID8 cells, or ID8 cells in which Akt isoforms were constitutively knocked down by stable expression of shRNAs delivered using a lentiviral vector system. ID8 cells expressing non-target shRNA were included as controls. Knockdown was confirmed with Western blot and immunofluorescence analysis. In a separate experiment, 1 × 10^6^ wild type ID8 cells were orthotopically injected under the ovarian bursa of WT, Akt 1^−/−^, Akt 2^−/−^, or Akt 3^−/−^ mice. In each experiment, mice were either sacrificed at 60d post tumor induction (PTI), or were allowed to progress to clinical signs of morbidity for survival analysis.

**Figure 2:**
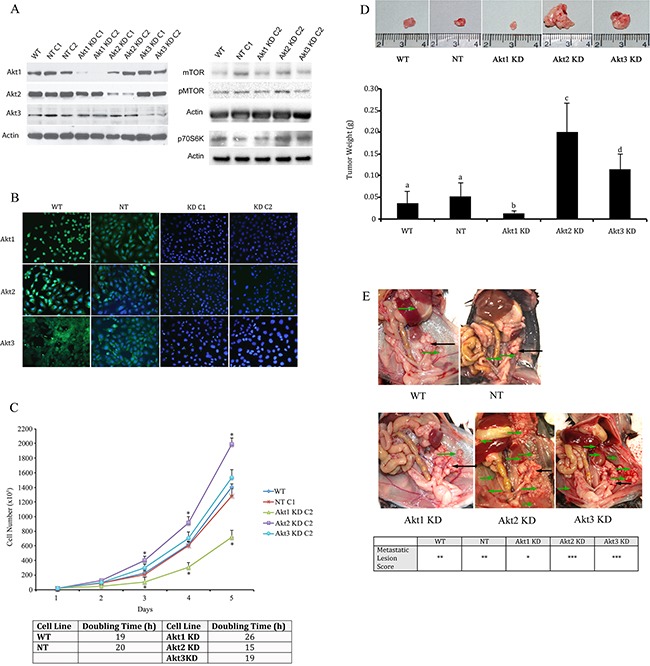
Knockdown of specific Akt Isoforms has variable effects on ovarian tumor development and metastatic disease **A.** Western blot analysis and **B.** immunofluorescence confirmed specific knockdown of Akt 1, Akt 2, and Akt 3 isoforms using lentiviral vector delivery of shRNA. **C.** Akt1 KD reduces, while Akt 2 KD elongates ID8 cell doubling time. 2×10^4^ ID8 cells were plated on 100mm cell culture dishes and cell number was counted using a hemocytometer every 24h for 5 days. * - statistically different from NT controls (p<0.05). Cell doubling times were quantified for each cell line and reported as the number of hours required for doubling of the cell population. **D.** At 60d post tumor induction, tumors were collected from mice injected with tumor cells in which the three Akt isoforms had been knocked down. Akt1 KD resulted in smaller tumors, while Akt2 knockdown led to larger tumors, with Akt3 KD resulting in an intermediate tumor size. (p<0.01). **E.** Akt1 knockdown also resulted in reduced metastatic disease compared to controls or other Akt isoform knockdowns. Primary tumors are shown with black arrows, while metastatic tumors are shown with green arrows. Metastatic tumors are sored as * < 3 visible tumors; ** 4-10 visible tumors; *** >, 10 visible tumors.

**Figure 3:**
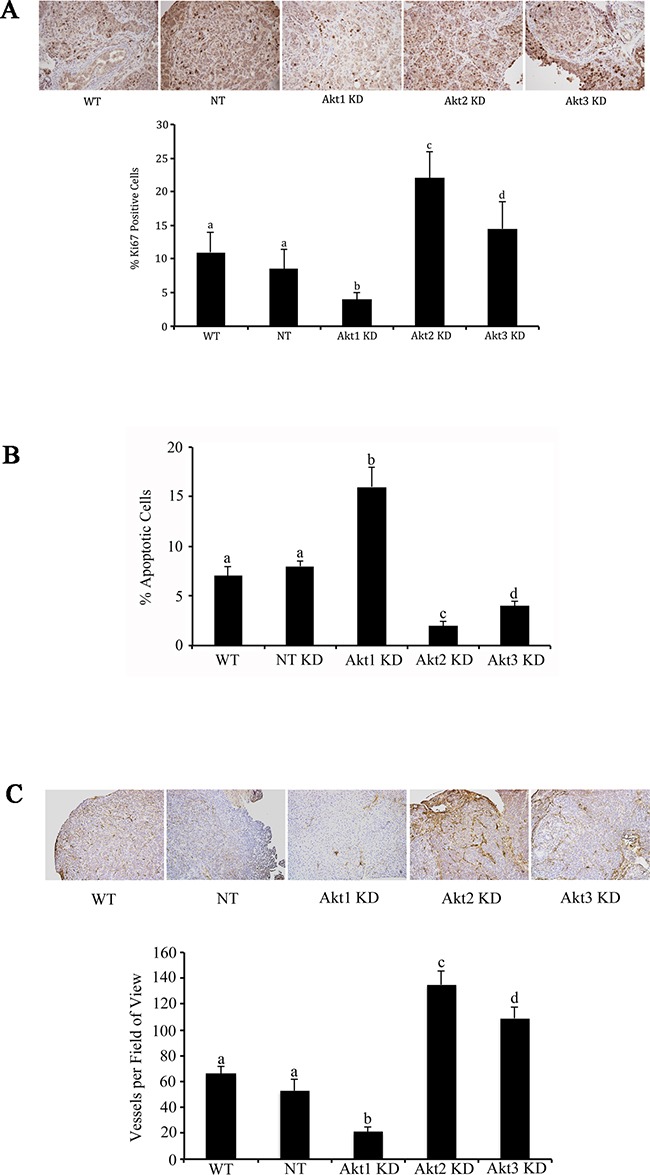
Akt isoform specific inhibition differentially affects tumor cell proliferation, apoptosis, and tumor vascularity Tumors were collected and fixed at 60d post tumor induction with control cells or cells with Akt isoforms knocked down. **A.** Ki67 immunostaining revealed a significant reduction in tumor cell proliferation following Akt1 knockdown and increased proliferation with knockdown of Akt2. **B.** Conversely, Akt 1 knockdown resulted in increased apoptosis, while Akt2 knockdown decreased ovarian tumor cell death. **C.** Ovarian tumors induced with Akt1 KD cells had reduced microvessel density, while Akt2 KD tumors had elevated vascularity. Tumors from Akt3 KD cells had an intermediate phenotype compared to the other two KD groups. Bars with different letters are statistically different (p<0.05).

**Figure 4:**
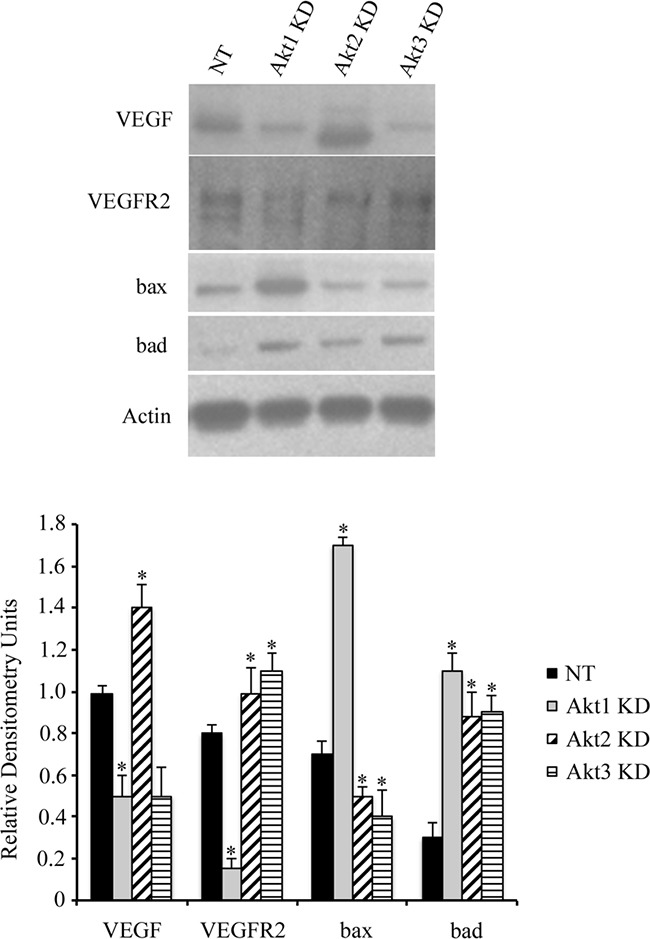
Akt isoform knockdown alters ovarian cancer cell signaling Akt1 knockdown resulted in decreased expression of VEGFR2 and increased levels of the pro-apoptotic proto-oncogenes bax and bad in lysates from tumors collected 60d post tumor induction. Conversely, Akt2 KD elevated expression of VEGF and its receptor while suppressing expression of bax. Tumors from Akt3 KD cells had increased VEGFR2 expression and reduced bax levels. * indicates statistical difference from NT control groups.(p<0.05). Graphs represent densitometric analysis of individual tumor lysates compared to Actin loading control from a minimum of n=4 animals per group.

### Akt knockdown affects survival in an isoform-specific manner

**Figure 5:**
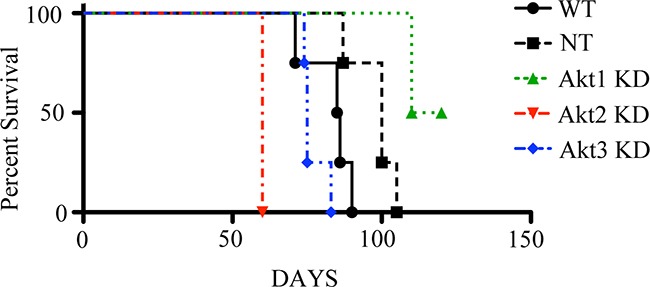
Tumor cell Akt isoform specific knockdown differentially impacts survival in a mouse model of epithelial ovarian cancer Tumors were induced with WT cells, or those in which Akt1, Akt2, or Akt3 were constitutively knocked down with lentiviral vectors expressing isoform-specific shRNAs or NT shRNA. Mice were allowed to progress until clinical signs of morbidity associated with ovarian cancer. Akt2 KD mice all had to be sacrificed by 60d post tumor induction. Akt1 KD mice survived the longest of all groups, while Akt3 KD mice were all euthanized by 82d post tumor induction. Log-rank analysis showed that isoform specific knockdown of Akt significantly (p<0.05) altered survival.

In a separate cohort of animals, tumors were induced with control or Akt1, Akt2, or Akt3 knockdown ID8 cells and animals were allowed to progress until signs of morbidity (accumulation of abdominal ascites) appeared, at which time the mice were euthanized to determine the effect of Akt knockdown on survival. Akt2 and 3 KD mice had reduced survival time (p<0.01), with Akt2 KD mice having the shortest survival (p<0.01), as all mice had to be euthanized on day 60 due to the presence of abundant abdominal ascites (Figure 5). It was apparent on the first day of experimental evaluation (day 60) that all Akt2 KD mice had significant abdominal distention, which was a termination criteria for the study. Akt1 KD mice, however, had the longest survival (p<0.01), with 50% of the animals still alive at day 120, when the experiment was terminated (Figure 5).

### Tumor induction in Akt Isoform knockout mice alters tumor growth and survival

**Figure 6:**
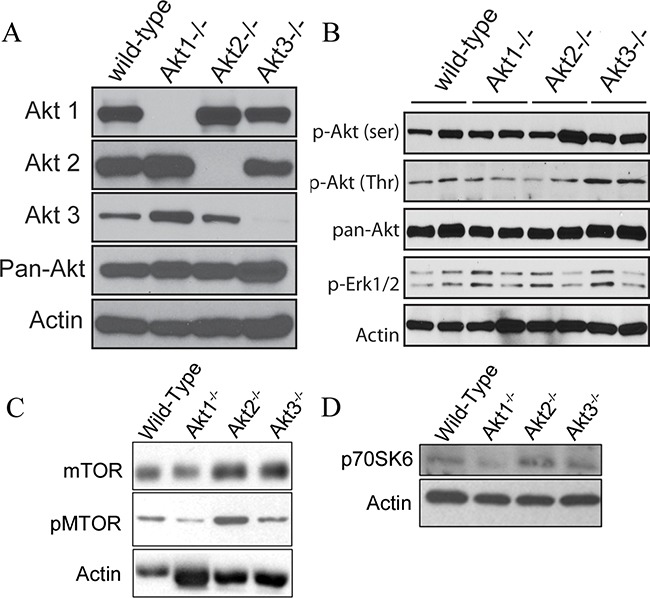
Akt signaling pathways are altered in ovarian tissue collected from WT, Akt1-, Akt2-, and Akt3-null mice **A.** Whole ovaries were collected from wild-type C57BL/6, Akt 1-, 2- or 3-null mice and subjected to Western blot analysis for each specific Akt isoform. **B.** Tumors collected from Akt 1, 2, and 3-null mice were probed for members of the AKT signaling family. C/D Tumor lysates from Akt 1, 2, and 3-null mice were evaluated for expression of members of the mTOR signaling pathway.

**Figure 7:**
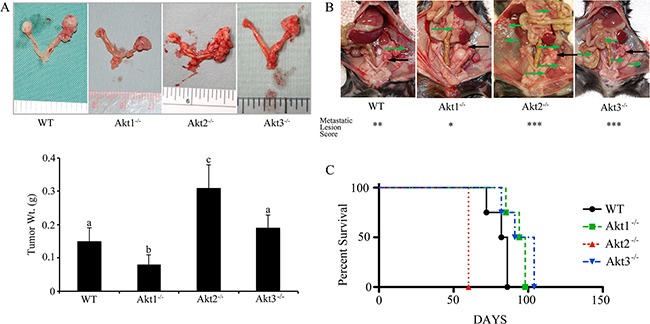
Ovarian tumors have a variable growth rate in Akt isoform deficient mice Wild type ID8 cells were injected into control WT mice, or mice null for Akt1, Akt 2, or Akt 3 isoforms. **A.** Tumors grown in Akt1^−/−^ mice were smaller, while those grown in Akt2^−/−^ mice were larger than other groups. Tumors grown in Akt3^−/−^ were not different from controls. Bars with different letters are statistically different (p<0.05). **B.** Akt1^−/−^ mice had fewer metastatic abdominal tumors at 60d post tumor induction, while Akt2^−/−^ and Akt3^−/−^ had a higher incidence of metastases, compared to WT controls. Primary tumors are shown with black arrows, while metastatic tumors are shown with green arrows. **C.** Akt2^−/−^ mice succumbed to disease earlier than the other groups (p<0.05).

To address the role of Akt isoforms in the tumor microenvironment, wild type ID8 cells were injected into Akt1, Akt2, and Akt3 null mice, in which the ovaries were confirmed to be null for the specific Akt isoform (Figure 6A). In tumors from Akt1 null mice, there was reduced expression and phosphorylation of pan-Akt (Figure 6B), as well as decreased activation of mammalian target of rapamycin (mTOR) and downstream member of the mTOR pathway p70SK6 (Figure 6C–6D). Conversely, tumors from Akt2 null mice had increased activation of the Akt pathway (Figure 6B) and elevated phosphorylation of mTOR and P70SK6 (Figure 6C–6D). Akt3 null tumors also exhibited increased Akt phosphorylation and activation of the mTOR pathway (Figure 6B–6D). Tumors from Akt1 null mice were significantly (p<0.05) smaller than all other groups at 60d post tumor induction (Figure 7a). Conversely, Akt2 null mice had tumors that were larger (p<0.05) than other groups, while tumors from Akt3 null mice were not different than controls (Figure 7a). When mice were evaluated for the presence of metastatic abdominal tumors, Akt 1^−/−^ mice had fewer abdominal tumors, compared to WT controls (Figure 7b). Conversely, Akt2^−/−^ and Akt3^−/−^ had increased metastatic tumors, compared to controls (Figure 7b). In a survival experiment, Akt 2 null mice died the earliest (p<0.05), with WT, Akt 1^−/−^ and Akt 3^−/−^ groups having similar survival (p>0.05) (Figure 7c).

### Isoform specific Akt inhibition differentially affects ovarian cancer cell viability, proliferation, and apoptosis

**Figure 8:**
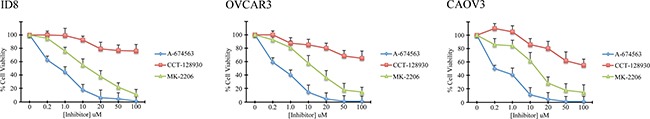
Akt isoform inhibition affects ovarian cancer cell viability WST-1 viability assay was performed on murine ID8 and human OVCAR3 and CAOV3 ovarian cancer cells following exposure of increasing doses of Akt inhibitors. The Akt1 selective inhibitor A-674563 induced the greatest reduction in viability.

Murine ID8 and human OVCAR and CAOV3 cells were treated with varying concentrations of the isoform specific inhibitors A-674563 (Akt1), CCT-128930 (Akt2), or MK-2206 (pan-Akt). Akt2 inhibition resulted in little reduction in viability of any of the ovarian cancer cell lines (Figure 8). Akt1 inhibition, however, decreased cell viability in the murine and ovarian cancer cells at dosages as low as 0.2μM (Figure 8a). Treatment of the cells with the pan-Akt inhibitor Mk-2206 resulted in an intermediate reduction in cell viability, compared to the Akt1 and Akt2 inhibitors (Figure 8)

## DISCUSSION

This paper demonstrates distinct roles for different Akt isoforms in regulating ovarian cancer progression. Inhibition of Akt1, both in the tumor cells and in our mice model, potently decreased ovarian tumor growth and decreased tumor proliferation, angiogenesis, and metastasis. Conversely, reducing the effects of Akt2 resulted in increased cell proliferation, accelerated tumor growth and elevated rates of metastasis. Ablation of Akt3, both in tumor cells and in the host often led to results that were intermediate between Akt1 and Akt2 knockdown. The phenotype of Akt3 knockdown appeared to be more similar to Akt2 than to Akt1 knockdown. A better understanding of the specific effects of the various Akt isoforms is critical to the development of targeted therapies with maximal efficacy in treating ovarian cancer.

Due to it's purported role in many cancers, Akt has become an interesting therapeutic target. Numerous studies have shown an association between high levels of Akt activity and advanced stage, high-grade serous adenocarcinoma [5, 20].

Although three distinct Akt isoforms have been identified - Akt1, Akt2, and Akt3 – their high degree of structural similarity led to the hypothesis that they have essentially redundant cellular functions. However, recent work suggests that there are important isoform-specific functional differences [13, 21–23]. Our data suggests that Akt1 is the main isoform responsible for ovarian cancer cell proliferation and protection against apoptosis and other studies have demonstrated an important role for Akt1 in ovarian cancer cell viability [24]. Interestingly, inhibition of Akt2 contributed to enhanced ovarian cancer cell proliferation and led to accelerated tumorigenesis *in vivo*. Elimination of Akt1 and 2 in the ovarian microenvironment had divergent effects on expression of members of the mTOR signaling pathway. Reduced Akt1 activity significantly inhibited expression and activation of the mTOR pathway, while Akt2 ablation resulted in enhanced mTOR signaling. mTOR activity is generally enhanced in epithelial ovarian cancer [25] and numerous clinical trials are currently in place using mTOR inhibitors in ovarian cancer. Others have shown opposing effects of Akt1 and 2 *in vitro* and have suggested that both isoforms compete for binding with p21 to regulate cell cycle kinetics [26]. In this paper, when Akt2 was knocked down, Akt1 was allowed to bind and phosphorylate p21, leading to enhanced cell proliferation. In a MMTV-ErbB2/Neu model of breast cancer, Akt1 ablation inhibited tumor formation, while Akt2 ablation enhanced mammary tumor growth [16]. In this paper, Akt1 deletion contributed to decreased proliferation and increased apoptosis in mammary tumors, similar to what we observed in the ovarian tumors in the present study. Akt2 is a potent regulator of the glucose transporter 1 (GLUT1), which facilitates uptake of glucose for cellular metabolism and is overexpressed in most cancer cells [27]. Targeted knockdown of Akt2 could lead to dysregulated glucose transport, enhancing cellular metabolism, although the isoform-specific effects on glucose transporter activation have not yet been measured.

Akt 2^−/−^ mice are known to be hyperglycemic and hyperinsulinemic [8], which could partially explain the enhanced tumorigenesis seen in these mice. Hyperglycemia is known to be associated with accelerated ovarian tumor growth and poorer outcome [28], and glucose inhibition can reduce ovarian tumorigenesis [29]. Similarly, increased insulin signaling due to elevated circulating insulin levels contributes to enhanced tumor growth [30], which along with hyperglycemic effects on tumor cells, suggests enhanced metabolism as a contributing factor to ovarian cancer progression. However, when Akt2 KD cells were injected into WT C57BL/6 mice, there was a similar increase in tumor growth rate, suggesting that influences other than metabolism are responsible.

Previous studies have postulated that inhibition of Akt1 can lead to an enhanced tumor cell invasiveness and more aggressive metastatic disease [31, 32]. In our study, we found that Akt1 inhibition reduced the presence of metastatic peritoneal tumors. The reduced metastatic potential that we observed could have been due to the decreased proliferative capacity and increased susceptibility to apoptosis that we observed. Many of the studies reporting increased invasiveness following Akt1 ablation were performed in breast cancer models, which rely on vascular metastasis. In ovarian cancer, the mechanism of metastasis is different, with transcoelomic dissemination of the tumor cells from the ovarian tumor directly into the peritoneal cavity. This different metastatic route and distinct environment could alter the cells' ability to divide and invade, compared to other models.

Most studies have focused on the roles of Akt1 and2 isoforms, with many fewer studying the role of Akt3. A recent paper reported levels of Akt3 similar to Akt1 and2 in lung tumors, but found that ablating Akt3 did not change tumor growth compared to controls [13]. In our study, Akt3 knockdown in tumor cells resulted in increased tumor size compared to controls, but to a lesser degree than that seen with knockdown of Akt2. Cristiano et al. [33] found high expression of Akt3 in primary ovarian tumors and demonstrated that knockdown of this isoform led to a decrease in ovarian cancer cell proliferation. In this paper, the reduced proliferation was seen in two cell lines that exhibited the highest levels of Akt3 expression, suggesting that Akt3 ablation may be effective in a subset of ovarian cancers. Recently, it has been shown that downregulation of Akt3 in a human breast cancer cell line increased migration *in vitro* and led to greater lung metastases *in vivo* [34]. In our *in vivo* model, we found an increase in tumor cell proliferation in Akt3 knockdown tumors compared to controls, but again this increase was significantly less than that seen in Akt2 KD tumors. One reason for this discrepancy may be that Akt3 has a lower expression in ID8 cells and thus ablating it might not have as great of an effect in this model.

The potential anti-cancer effects of Akt inhibition has led to the development of a number of Akt targeted therapies and several of these are currently in clinical trials. The pan-Akt inhibitor MK-2206 inhibits all three Akt isoforms and currently is being used in a number of NCI-supported clinical trials for pancreatic, colorectal, breast, and prostate cancers (National Cancer Institute, 2015). MK-2206 has been shown to augment the efficacy of chemotherapy drugs in ovarian cancer cells [35, 36]. The Akt2 inhibitor CCT-128930 has also been tested in cancer cell lines and resulted in cell cycle arrest, increased Annexin localization and autophagy markers, although these effects were seen at very high concentration of inhibitor [37]. Akt1-specific inhibitors have also been developed recently. A-674563 is an ATP competitive inhibitor with high affinity for Akt1 [38]. Similar to the results seen in this study, specific inhibition of Akt1 induced a significant increase in apoptotic death of renal cell carcinoma cells both *in vitro* and *in vivo* [39]. A recent study also demonstrated isoform specific effects in lung cancer, where the Akt1 inhibitor A-674563 more potently affected cell survival than the pan-Akt inhibitor MK-2206 [40]. Interestingly, the Akt1 inhibitor A-674563 has also been shown to reduce survival of endothelial colony forming cells and inhibit vasculogenesis *in vitro* [41], which corresponds to the reduced microvessel density that we saw in ovarian tumors induced with Akt1 KD cells. Akt1 is an important regulator of VEGF mediated angiogenesis [42] and Akt1 null mice exhibit vascular abnormalities, with increased apoptotic susceptibility in endothelial and vascular smooth muscle cells [43, 44]. As such, isoform-specific inhibition may also result in an anti-angiogenic inhibition of tumor growth, in addition to disrupted tumor cell signaling and viability. Limited information is available on the role of Akt2 and 3 on regulating angiogenesis, and this would be an important area of further study. Akt inhibitors are also being tested in clinical trials in women with ovarian cancer. Perifosine, an oral pan-Akt inhibitor, demonstrated a partial response in 1 patient, and stable disease in 3 patients in a Phase I trial [45]. In a recently completed Phase II study evaluating the efficacy of the pan-Akt inhibitor MK2206 in recurrent platinum resistant ovarian cancer (NCT01283035), 4/5 patients reported stable disease, while 1/5 had progressive disease. A Phase I trial with another pan-Akt inhibitor GSK795, also demonstrated moderate results, with stable disease in 16% of patients and some evidence of tumor shrinkage [46]. The results from the current study suggest that a contributor to the modest efficacy of the pan-Akt inhibitors in ovarian cancer is the incongruous effects of the individual Akt isoforms. By administering an inhibitor that affects all Akt isoforms, there may be a muting of therapeutic efficacy. Conversely, by using focused inhibition of Akt1, there would be refined and enhanced anti-tumor effects through inhibition of Akt/mTOR signaling, tumor cell proliferation and survival and metastasis.

In this study, we manipulated Akt isoform levels both in the cells used to induce tumor formation as well as in the host. Interestingly, we found similar effects on tumor progression, suggesting that Akt isoforms have distinct and important functions both within the tumor cells and within the tumor microenvironment. These data suggest that treatment with a pharmacological inhibitor of Akt1 may have maximal effectiveness as the drug would reach both tumor cells and the tumor microenvironment. The tumor microenvironment is integral to tumor progression as it contributes to essential processes such as paracrine survival signaling and angiogenesis. Aside from well-established effects on maintaining cell viability within the tumor directly, inhibition of Akt signaling has been reported to have indirect anti-tumor effects within the microenvironment as it reduces responsiveness to tumor pro-angiogenic signals [47] and alters paracrine influences on cellular proliferation, survival, and invasion [48]. As such, systemic exposure to isoform-specific Akt inhibition that targets both the tumor cells and tumor microenvironment may be particularly effective.

Data from this study illustrate distinct and potentially opposing effects of the specific Akt isoforms in epithelial ovarian cancer progression. Careful attention must be paid to Akt-mediated therapies as pan-Akt inhibition may result in muted effects due to the manipulation of isoforms that have differing influences on tumor response. Our study suggests that specific disruption of Akt1 may be preferable to pan-Akt inhibition for the treatment of ovarian cancer.

## MATERIALS AND METHODS

### Cell culture

The ID8 murine ovarian surface epithelial cell line (generously donated from Drs. Paul Terranova and Kathy Roby, Kansas State University) was cultured in Dulbecco's Modified Eagles Medium (DMEM) supplemented with 10% FBS and 1% antibiotic/antimycotic (Gibco) and maintained at 37°C and 5% CO_2_. Human epithelial ovarian cancer cell lines OVCAR3 and CAOV3 were purchased from the American Type Culture Collection (ATCC, Manassas VA, USA) and cultured in RPMI with 20% FBS and 1% antibiotic/antimycotic (OVCAR3) or DMEM with 10% FBS and 1% antibiotic/antimycotic (CAOV3). OVCAR3(5) and CAOV3 [49] express all Akt isoforms and Akt phosphorylation is involved in proliferation in these cell lines.

### Knockdown of Akt isoforms in ID8 cells using lentiviral vectors expressing isoform specific shRNAs

pLKO.1-puro vectors encoding murine Akt1, Akt2, Akt3 and scrambled (non-target) shRNA were generated as described previously [50]. Pseudotyped lentiviral vectors were generated by calcium phosphate-mediated co-transfection of the plasmids psPAX2 (6.5 μg), pCMV-VSV-G (3.5 μg), and pLKO.1-puro-shRNA (10 μg) into HEK293T cells seeded in a 10 cm dish. Medium was removed after 16 hr and replaced with 5 ml of fresh DMEM supplemented with 10% FBS, 1% L-Glutamine 1% Penicillin-Streptomycin. After 48 hr, virus containing supernatants were collected, 0.45 M filtered and stored at −80°C until used. ID8 cells were transduced with 250ul of virus-containing medium in the presence of 8 g/ml polybrene (Sigma) and clonally selected using 1.5μg/mL of puromycin. Ten clones were randomly selected and screened for Akt isoform specific knockdown by immunofluorescence and Western blot analysis. Two different clones for each isoform were used for *in vitro* and *in vivo* experiments. Non-target (NT) clones were selected based on wildtype Akt1, 2 and 3 expression levels. For cell growth studies using Akt isoform specific ID8 knockdowns, 2×10^4^ WT, NT, Akt1 KD, Akt2 KD, or Akt3 KD cells were seeded on 100 mm culture dishes at day 0. Cells in three dishes of each cell line were counted every 24h for 5 days using a hemocytometer.

### Immunofluorescence microscopy

ID8 clonal cell lines were screened for specific Akt isoform knockdown using immunofluorescence microscopy. Cells were plated onto coverslips and fixed with 3.7% paraformaldehyde-PBS for 10 mins at room temperature. Cells were permeabilized using 0.2% Triton X in PBS, blocked in 4% BSA-PBS and incubated overnight at 4°C with Akt specific antibodies (1:100; Akt1 and 2 antibodies were from Cell Signaling, Akt3 antibody was from Millipore). Cells were washed with PBS three times and incubated with an Alexa Fluor 488-conjugated secondary antibody (1:100; Invitrogen/Life Technology) for 1 hour at room temperature. Following a final wash in PBS, coverslips were mounted using Prolong Gold with DAPI (Invitrogen) and captured using a Carl Zeiss Axio 154 Observer A1 inverted fluorescence microscope.

### Western blot

Monoclonal antibodies specific for Akt isoforms 1, 2 and 3, phospho-Akt (Thr308), phospho-Akt (Ser473) and pan-Akt were purchased from Cell Signaling Technology. Polyclonal antibodies to VEGF (Santa Cruz), VEGFR2 (Abcam), bax (Santa Cruz), bad, mTOR, pMTOR, and p70SK6 (Cell Signaling) were used to determine changes in tumor cell signaling following isoform specific knockdown or in isoform specific null mice. Equal protein loading was verified using anti-β-actin antibody from Santa Cruz Biotechnologies. Protein was collected from ID8 cells with specific Akt isoform knockdown using RIPA buffer containing a cocktail of protease and phosphatase inhibitors (Sigma). Cell lysates were separated by 12% SDS-PAGE gel electrophoresis and transferred to a PVDF membrane. Membranes were blocked with 4% BSA in PBST and incubated overnight at 4°C with primary antibody (1:1000 p-Akt (Ser), p-Akt (Thr), pan-Akt, VEGF, mTOR, pMTOR, p70SK6; 1:600 dilution VEGFR2, bax, bad). Secondary anti-rabbit, anti-mouse, and anti-goat HRP-conjugate antibodies (1:2000, Invitrogen) were added to the membrane for 1 hour at room temperature and proteins were detected using Western Lightning Plus Chemiluminescence (Perkin-Elmer). Images were captured using X-ray film (VWR).

### Ethics

Animals were housed and cared for in strict accordance with the Canadian Council of Animal Care (CCAC) guidelines. The animal use protocol was approved by the Animal Care Committee (ACC) of the University of Guelph. All efforts were made to minimize suffering.

### Mouse models

Mouse models were designed to determine the effect of Akt isoform-specific knockdown in ID8 cancer cells used for tumor induction or to determine the effect of WT ID8 tumor cell induction of ovarian cancer progression in Akt1, Akt2, or Akt3 null mice. Wild type C57BL/6 mice were purchased from Charles River Laboratories, while Akt1^−/+^ and Akt2^−/+^ mice were purchased from Jackson Laboratory (USA) and bred to obtain homozygous Akt1^−/−^ and Akt2^−/−^ knockout mice. Akt3^−/−^ mice were generously provided by Dr. Morris Birnbaum (University of Pennsylvania) [8]. All mice were housed at the Central Animal Facility at the University of Guelph. To evaluate the role of individual Akt isoforms on EOC, we used an orthotopic, syngeneic mouse model for EOC described previously [19, 51, 52] in which we injected 1 × 10^6^ spontaneously transformed murine ovarian surface epithelial cells derived from C57BL/6 mice (ID8) in which Akt1, Akt2, or Akt3 isoforms had specifically been knocked down using shRNA under the ovarian bursa. Methods associated with Akt isoform knockdown are described above. We also performed orthotopic injections of 1 × 10^6^ WT ID8 cells under the bursa of Akt1^−/−^, Akt2^−/−^, or Akt3^−/−^ mice to determine the effect of loss of Akt isoforms within the tumor microenvironment. In both animal models, mice were euthanized at either 60 days post-tumour induction or at end point of disease (Figure 1). Primary tumors were weighed, ascites volume was recorded and number and location of metastasis was quantified using the following scoring system: no observable lesions (-), 1-2 local lesions (*), 3-10 lesions throughout the abdomen (**) and greater than 10 lesions (***).

### Mouse survival

End point of disease were assessed on the basis of visible abdominal distention due to ascites accumulation, increase in weight gain of 20% of their pre-tumour induction body weight or any clinical signs of morbidity. Half of the mice were euthanized at 60 days post tumour induction and the rest were euthanized at disease endpoint. No mice survived past 120 days. Both PBS and ID8 injected ovaries were collected and histologically examined. Half the tissues were flash frozen in liquid nitrogen and the other half were fixed in 10% neutral buffered formalin and paraffin embedded.

### Immunohistological staining for proliferation and apoptosis

Paraffin embedded tissues were sectioned and subject to immunohistochemical staining as was described previously (Linnerth-Petrik et al., 2014). Proliferating cells were manually counted using an anti-Ki67 antibody (Abcam) as described previously (Linnerth-Petrik et al., 2014) and images were captured using a brightfield microscope. Three images were captured per tissue section, with a minimum of 3 mice per group used for quantification.

TUNEL staining was performed on end-point paraffin-embedded ovarian tumor tissue sections using the In Situ Cell Death Detection Kit, POD (Roche Diagnostics) according to the manufacturer's protocol. TUNEL-positive nuclei were counted as described above.

### Akt isoform inhibition in ovarian cancer cells

Murine (ID8) and human (OVCAR3, CAOV3) epithelial ovarian cancer cells were exposed to Akt isoform specific inhibitors in culture. To determine isoform-specific inhibitor effects on cell viability, cells were exposed to a variety of concentrations (0, 0.2, 1.0, 10, 20, 50, and 100μM) of Akt1 (A-674563; Sellekchem); or Akt2 (CCT-128930; Sellekchem) inhibitors, or the pan-Akt (MK-2206; Sellekchem) inhibitor for 72hr and then subjected to a WST-1 viability assay. Briefly, 1×10^3^ ID8, OVCAR3, or CAOV3 cells were seeded and incubated in 96-well culture vessels in 99uL of Media. After 24hr, either 1ul DMSO or increasing concentrations of inhibitors were added. Media and inhibitors were replaced every 24hr until 72hr when WST-1 reagent was added at 37°C for 2hr and optical density was determined at 450nm using a microplate reader.

### Statistics

Statistical significance was determined using a two way ANOVA followed by Tukey's post-hoc test to determine differences between groups. For evaluation of survival, the log-rank test was performed by GraphPad Prism v6 software (GraphPad Software, La Jolla, CA). Unless stated otherwise, statistical significance was considered at p<0.05. For animal experiments, each group consisted of a minimum of n=5.
